# Emerging Therapeutic Activity of *Davallia formosana* on Prostate Cancer Cells through Coordinated Blockade of Lipogenesis and Androgen Receptor Expression

**DOI:** 10.3390/cancers12040914

**Published:** 2020-04-08

**Authors:** Po-Fan Hsieh, Wen-Ping Jiang, Shih-Yin Huang, Praveenkumar Basavaraj, Jin-Bin Wu, Hui-Ya Ho, Guan-Jhong Huang, Wen-Chin Huang

**Affiliations:** 1 Graduate Institute of Biomedical Sciences, School of Medicine, China Medical University, Taichung 404, Taiwan; 2 Department of Urology, China Medical University Hospital, Taichung 404, Taiwan; 3 School of Chinese Pharmaceutical Sciences and Chinese Medicine Resources, College of Chinese Medicine, China Medical University, Taichung 404, Taiwan; 4 Nihon Pharmaceutical University, 10281, Komuro, Ina-machi, Kitaadachi-gun, Saitama 3620806, Japan; 5 Jen Li Biotech Company Ltd., Taiping District, Taichung 411, Taiwan

**Keywords:** *Davallia formosana*, anti-prostate cancer efficacy, SREBP-1/FASN/lipogenesis, AR

## Abstract

Background: Prostate cancer (PCa) is the most prevalent malignancy diagnosed in men in Western countries. There is currently no effective therapy for advanced PCa aggressiveness, including castration-resistant progression. The aim of this study is to evaluate the potential efficacy and determine the molecular basis of *Davallia formosana* (DF) in PCa. Methods: LNCaP (androgen-sensitive) and C4-2 (androgen-insensitive/castration-resistant) PCa cells were utilized in this study. An MTT-based method, a wound healing assay, and the transwell method were performed to evaluate cell proliferation, migration, and invasion. Intracellular fatty acid levels and lipid droplet accumulation were analyzed to determine lipogenesis. Moreover, apoptotic assays and in vivo experiments were conducted. Results: DF ethanol extract (DFE) suppressed proliferation, migration, and invasion in PCa cells. DFE attenuated lipogenesis through inhibition of the expression of sterol regulatory element-binding protein-1 (SREBP-1) and fatty acid synthase (FASN). Moreover, DFE decreased androgen receptor (AR) and prostate-specific antigen (PSA) expression in PCa cells. We further showed the potent therapeutic activity of DFE by repressing the growth and leading to apoptosis of subcutaneous C4-2 tumors in a xenograft mouse model. Conclusions: These data provide a new molecular basis of DFE in PCa cells, and co-targeting SREBP-1/FASN/lipogenesis and the AR axis by DFE could be employed as a novel and promising strategy for the treatment of PCa.

## 1. Introduction

Chinese/Oriental medicine is a healing and herbal system that has been built on a foundation for a long history, for the successful treatments of infectious and chronic diseases, and even cancers. *Davallia formosana* (DF) is one of the native ferns, which is specifically cultivated in Taiwan and has been considered a Chinese medicine. Significantly, DF and DF ethanol extract (DFE) are applied for the treatment of bone diseases, including rheumatoid arthritis [1], bone fractures, and osteoporosis [2,3]. However, the clinical benefits and the underlying molecular mechanisms of DFE in prostate cancer remain to be explored.

Prostate cancer (PCa) cells are initially and typically in need of androgens and the androgen- signaling axis for growth, survival, and progression. One of the most challenging and unsolved clinical issues is that PCa progresses from androgen-dependent to androgen-refractory/castration-resistant prostate cancer (CRPC), which is recognized as a lethal disease. The androgen signaling axis is predominately mediated by the androgen receptor (AR). AR is a key driver and plays a critical role in regulation of PCa development and CRPC progression [4,5]. Blockade of the androgen/AR-mediated signaling pathways by androgen deprivation therapy (ADT) was determined to be an effective approach for the treatment of PCa and remains a key component of current therapy regimens [6]. However, most PCa patients who receive ADT eventually progress to CRPC, and even the new-generation AR-targeted agents provide limited survival benefits [7]. Thus, developing a new and effective therapeutic strategy to treat and overcome this fatal progression is a compelling need.

Aberration of metabolism/biosynthesis is one of the emerging hallmarks in cancer cells [8]. Research data also indicate that activation of lipogenesis could induce and promote PCa cell growth, survival, and aggressiveness [9,10,11]. Sterol regulatory element-binding protein-1 (SREBP-1), a transcriptional factor, mainly controls expression of genes associated with lipogenesis and fatty acid/lipid homeostasis [12]. SREBP-1 would bind with sterol regulatory elements (SRE) which are found in the 5’-flanking promoter regions of genes encoding enzymes for de novo lipogenesis [13,14]. Specifically, SREBP-1 transcriptionally induces expression of fatty acid synthase (FASN), a lipogenic check point, and a rate-limiting enzyme for de novo biosynthesis of long-chain fatty acids [15]. Importantly, increased SREBP-1 expression has been demonstrated to be positively correlated with aggressive PCa pathologic features and lethal CRPC progression in patients [11,16]. Additionally, overexpression of FASN was also associated with cancer progression and poor prognosis in colorectal [17], breast [18], and prostate [19,20] cancers. Attenuation of SREBP-1 or FASN expression leads to inhibition of cell growth and progression, and induction of apoptosis in PCa and other cancers [21,22,23,24]. Collectively, these clinical and experimental data provide rationale for targeting SREBP-1 and/or FASN as an attractive and potential therapeutic strategy in the management of malignant PCa.

In this study, we evaluated the anti-PCa efficacy of DFE and determined the molecular basis of DFE in androgen-dependent LNCaP and castration-resistant C4-2 PCa cells. DFE significantly inhibited cell growth and suppressed migratory and invasive capabilities in both LNCaP and C4-2 cells. Furthermore, DFE decreased messenger RNA (mRNA) and protein expression of SREBP-1 and FASN in PCa cells. Through inhibiting the expression of key genes associated with lipogenesis (SREBP-1 and FASN), DFE reduced the amounts of intracellular fatty acids and lipid droplets in PCa cells. Intriguingly, DFE also impaired the expression of AR and prostate-specific antigen (PSA) in LNCaP and C4-2 cells. By coordinated blockade of SREBP-1/FASN/lipogenesis and the AR axis, DFE triggered programmed cell death via activation of the caspase-dependent apoptotic pathway in PCa cells and subcutaneous C4-2 tumors in mice. Our findings offer new insight into the underlying molecular basis of DFE in PCa and a novel promising therapeutic opportunity to eradicate PCa malignancy.

## 2. Results

### 2.1. DFE Suppresses PCa Cell Growth, Migration, and Invasion In Vitro

**Figure 1:**
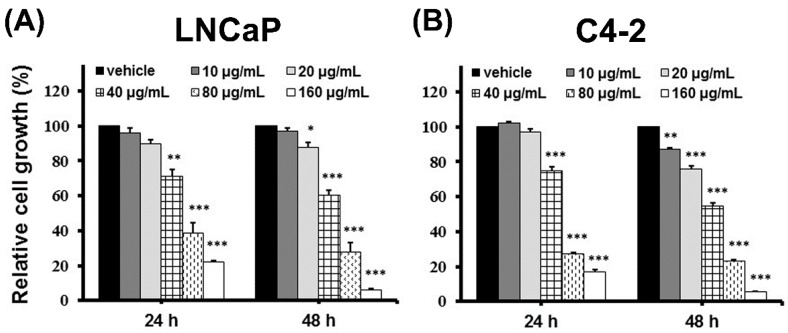
*Davallia formosana* (DF) ethanol extract (DFE) suppresses prostate cancer (PCa) cell growth. (**A**) LNCaP and (**B**) C4-2 cells were treated with vehicle or DFE (10, 20, 40, 80, and 160 µg/mL) for 24 and 48 h. PCa cell growth was determined by MTT analysis. The relative cell growth (%) was shown as 100 in vehicle-treated PCa cells at each time point and each cell line. Data represented as the mean ± SD of three independent experiments. * *p* < 0.05, ** *p* < 0.01, *** *p* < 0.001.

DFE, a Chinese herbal medicine, has been well employed to cure several bone disorders with low cytotoxicity [1,2,3]. However, the therapeutic efficacy of DFE on PCa is still unknown. To explore the potential for clinical benefits elicited by DFE, androgen-dependent LNCaP and castration-resistant C4-2 cells, two PCa cell lines with various biological heterogeneities [25], were treated with various amounts of DFE or vehicle (0.06% DMSO) followed by the functional analyses, including cell growth, migration, as well as invasion assays. DFE suppressed the growth of LNCaP and C4-2 cells in time (24 and 48 h)- and dose (10, 20, 40, 80, and 160 µg/mL)-dependent patterns (Figure 1A,B). The IC_50_ (half maximal inhibitory concentrations) of DFE for LNCaP and C4-2 cells were 52.5 and 46.0 µg/mL (at 48 h), respectively.

**Figure 2:**
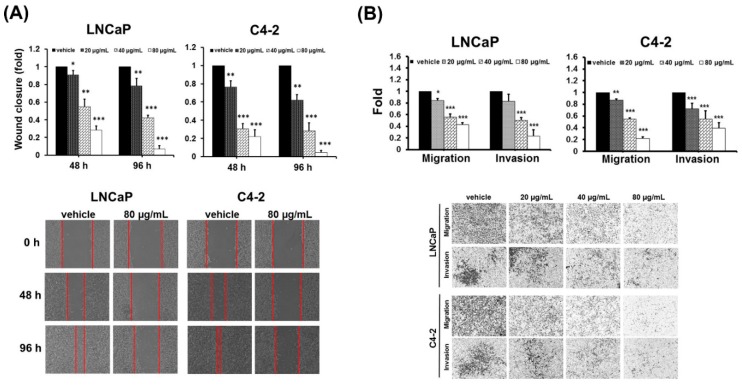
DFE reduces migration and invasion in PCa cells. (**A**) A wound healing assay. LNCaP and C4-2 cells were exposed to DFE (20, 40, and 80 µg/mL) or control vehicle. Wound closure was analyzed by the migratory distance at 48 and 96 h, respectively. Results represented as the mean ± SD of three independent experiments. * *p* < 0.05, ** *p* < 0.01, *** *p* < 0.001. Representative images of wound closure in PCa cells exposed to DFE (80 µg/mL) or vehicle at different time points were shown (bottom panel). (**B**) The migration and invasion of LNCaP and C4-2 cells were determined by the transwell method. The relative migration or invasion was defined as 1.0 (Fold) in the vehicle-treated cells. Data represented the mean ± SD of three independent experiments. * *p* < 0.05, ** *p* < 0.01, *** *p* < 0.001. The images of the migration and invasion of LNCaP and C4-2 cells exposed to vehicle or DFE at 48 h were shown (bottom panel).

Next, the effects of DFE on the PCa migratory and invasive potentials, the hallmarks of progressive cancer cells, were investigated. A wound healing assay was first applied to evaluate the migratory ability of PCa cells affected by DFE. As shown in Figure 2A (top and bottom panels), DFE led to a significant inhibition of wound closure compared to a vehicle group in PCa cells after 48 and 96 h treatments. Subsequently, migration and invasion of PCa cells affected by DFE were examined using the transwell method. DFE significantly suppressed the capabilities of migration and invasion in both LNCaP and C4-2 cells in a dose-dependent manner (20, 40, and 80 µg/mL) (Figure 2B, top and bottom panels). Taken together, the data of the functional analyses indicate that DFE led to suppression of cell growth, migration, and invasion in PCa cells.

### 2.2. DFE Inhibits SREBP-1/FASN and AR/PSA Expression in PCa Cells

**Figure 3:**
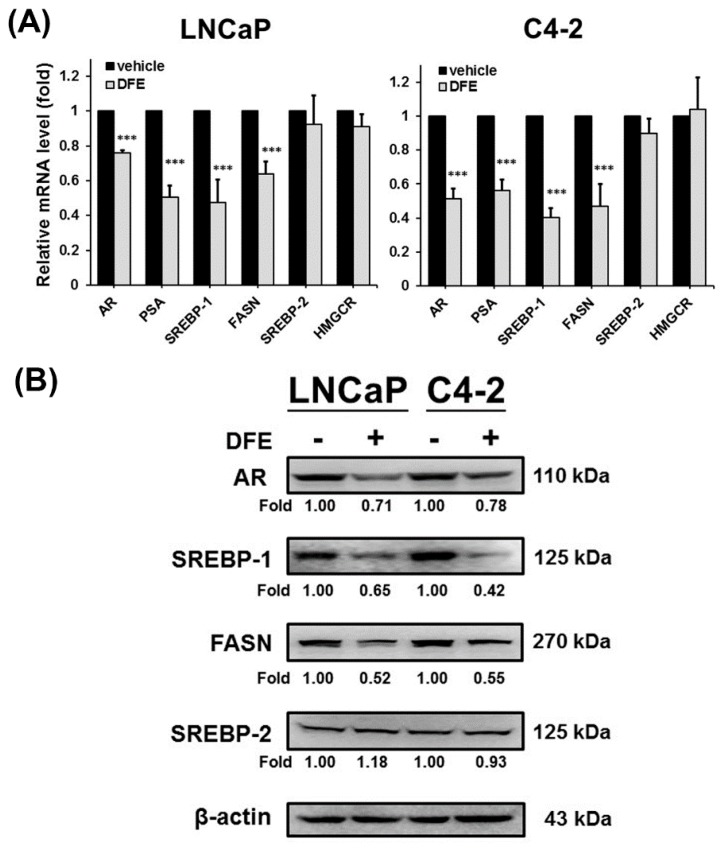
DFE inhibited sterol regulatory element-binding protein-1 (SREBP-1)/fatty acid synthase (FASN) and androgen receptor (AR)/prostate-specific antigen (PSA) expression in LNCaP and C4-2 cells. (**A**) DFE inhibited the messenger RNA (mRNA) expression of AR/PSA and SREBP-1/FASN, but did not significantly affect SREBP-2 and 3-hydroxy-3-methyl-glutaryl-coenzyme A reductase (HMGCR) mRNA expression in PCa cells. The relative mRNA level was defined as 1.0 (fold) in the vehicle-treated cells. Data were normalized by β-actin mRNA expression and shown as the mean ± SD of triplicate assays with three independent experiments. *** *p* < 0.001. (**B**) DFE reduced the protein amounts of AR, SREBP-1, and FASN in LNCaP and C4-2 cells. β-actin was used as a loading control. Data were normalized by β-actin protein expression. The relative fold was defined as 1.00 in the vehicle-treated LNCaP or C4-2 cells, respectively.

Blocking SREBP-1/FASN/lipogenesis and the AR axis have demonstrated to suppress PCa growth and progression [16,22,26,27]. To further evaluate if DFE suppresses PCa cell growth, migration and invasion would be associated with these metabolic and signaling pathways; RT-qPCR and Western blot analyses were performed to examine the expression of genes linked to the AR axis and lipogenesis. The results of RT-qPCR showed that DFE significantly decreased the mRNA expression of AR and its downstream regulated gene, prostate-specific antigen (PSA), in LNCaP and C4-2 cells (Figure 3A). Furthermore, DFE inhibited SREBP-1 and FASN mRNA expression but did not significantly change SREBP-2 (a key factor for cholesterogenesis) and 3-hydroxy-3-methyl-glutaryl-coenzyme A reductase (HMGCR) expression, a downstream target gene of SREBP-2, in LNCaP and C4-2 cells (Figure 3A). Moreover, consistent with the effects of DFE on the mRNA expression, the results of Western blotting demonstrated that the protein levels of AR, SREBP-1, and FASN, but not SREBP-2, were inhibited by DFE in PCa cells (Figure 3B). The data of gene expression collectively indicate that DFE inhibited the expression of SREBP-1/FASN and AR/PSA in PCa cells.

### 2.3. DFE Decreases Lipogenesis in PCa Cells

**Figure 4:**
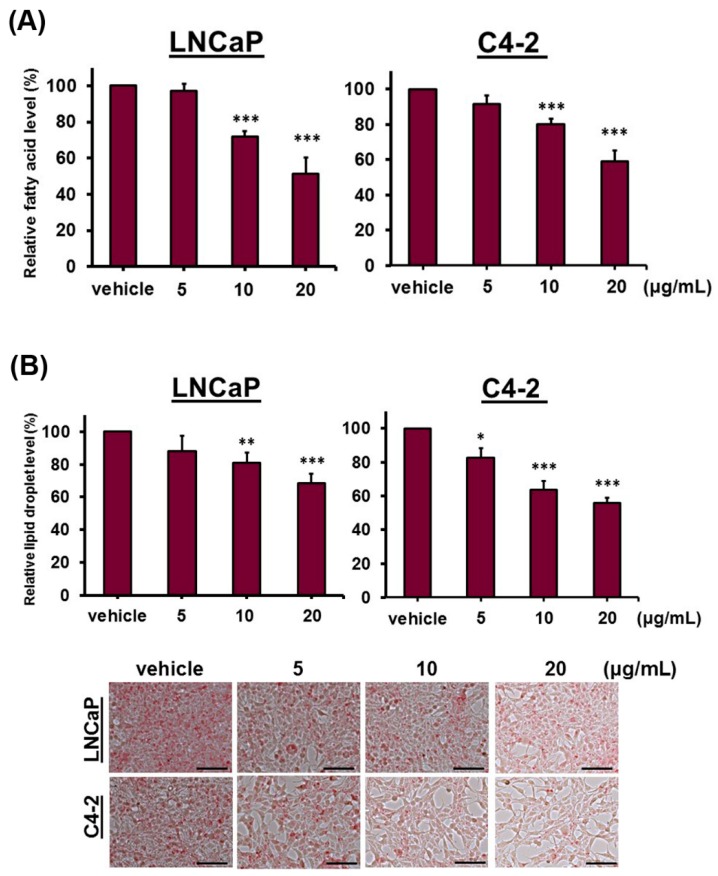
DFE attenuates lipogenesis in PCa cells. (**A**) DFE significantly decreased the intracellular fatty acid levels in LNCaP and C4-2 cells assayed by a Fatty Acid Quantification Kit. Data were shown as the mean ± SD of two independent assays, and normalized to PCa cell numbers. * *p* < 0.05, ** *p* < 0.01, *** *p* < 0.001. (**B**) DFE reduced lipid droplet accumulations in PCa cells examined by an Oil Red O staining assay. Data were shown as the mean ± SD of triplicate analyses in two independent experiments, and normalized to PCa cell numbers. *p < 0.05, **p < 0.01, ***p < 0.001 (top panel). The lipid droplet accumulation images in PCa cells were shown (bottom panel). Scale bars = 100 µm.

DFE reduced the expression of key factors, SREBP-1 and FASN, which regulated lipogenesis; we next performed staining assays and quantification analyses of lipogenesis to assess the levels of intracellular fatty acids and lipid droplets in PCa cells treated with DFE. As shown in Figure 4A, DFE affected the fatty acid levels in both LNCaP and C4-2 cells with a concentration-decreased pattern compared to the vehicle group. Additionally, DFE also significantly diminished the levels (Figure 4B, top panel) and staining (Figure 4B, bottom panel) of lipid droplets in LNCaP and C4-2 cells. The results of lipogenic experiments indicate that by inhibition of SREBP-1 and FASN expression, DFE decreased the fatty acid levels and lipid droplet accumulations in both LNCaP and C4-2 cells.

### 2.4. DFE Induces Caspase-Dependent Apoptosis Leading to PCa Cell Death

**Figure 5:**
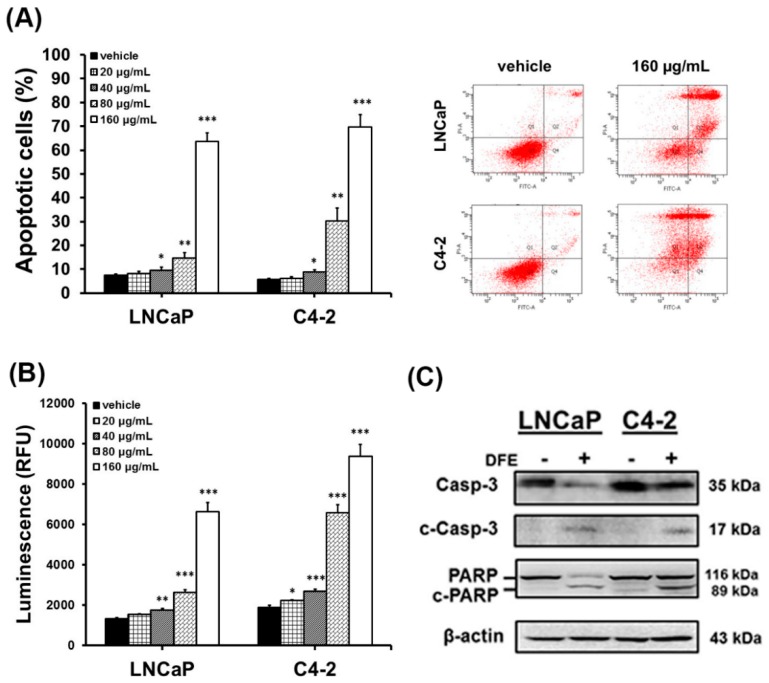
DFE induces caspase-dependent apoptosis leading to PCa cell death. (**A**) LNCaP and C4-2 cells were exposed to vehicle or DFE for 24 h. Apoptotic cells (%) were subsequently measured by Annexin V/ propidium iodide (PI) staining flow cytometry-based assay. Data represented the mean ± SD of triplicate analyses. *p < 0.05, **p < 0.01, ***p < 0.001. (**B**) The enzymatic activity of caspase-3 was induced by DFE in a dose-dependent manner. Luminescence (RFU: Relative Fluorescence Unit) was determined by Caspase-Glo^®^ 3 Assay System (Promega). Data represented as the mean ± SD of triplicate assays. (**C**) The protein expression levels of caspase-3 and poly (ADP-ribose) polymerase (PARP) were determined by Western blotting in PCa cells exposed to vehicle (−) or DFE (160 µg/mL; +). DFE decreased caspase-3 (Casp-3; 35 kDa) and PARP (116 kDa), and increased cleaved (c)-Casp-3 (17 kDa) and c-PARP (89 kDa) in LNCaP and C4-2 cells. β-actin was a loading control.

Several studies demonstrated that impairment of SREBP-1/FASN/lipogenesis and the AR axis leads to apoptosis in cancer cells [21,22,23,28]. Subsequently, we investigated if DFE led to cell death and induced the caspase-dependent apoptosis in PCa cells. DFE increased the numbers (%) of apoptotic PCa cells in a dose-dependent manner determined by Annexin V-Fluorescein isothiocyanate (FITC)/propidium iodide (PI) staining flow cytometry-based assay (Figure 5A). Significantly, apoptotic cells were 63.70% ± 3.58% (LNCaP) and 69.63% ± 5.35% (C4-2) treated by DFE (160 µg/mL, 24 h), respectively (Figure 5A). The vehicle-treated apoptotic PCa cells were only 7.47% ± 0.55% (LNCaP) and 5.64% ± 0.45% (C4-2) (Figure 5A). Furthermore, the catalytic activity of caspase-3, as well as the protein expression of caspase-3 and poly (ADP-ribose) polymerase (PARP), were determined to assess if caspase-dependent apoptosis was activated by DFE in PCa cells. DFE induced the enzymatic activity of caspase-3 in a concentration-dependent manner in LNCaP and C4-2 cells (Figure 5B). Besides, DFE treatment decreased the protein levels of both caspase-3 and PARP but induced the protein expression of cleaved (c)-caspase-3 (17 kDa) and c-PARP (89 kDa) in LNCaP and C4-2 cells assayed by Western blotting (Figure 5C). The results of the apoptotic study suggest that DFE treatment promoted cell death via the induction of caspase-dependent apoptosis in PCa cells. 

### 2.5. DFE Displays Anti-PCa Activity In Vivo

**Figure 6:**
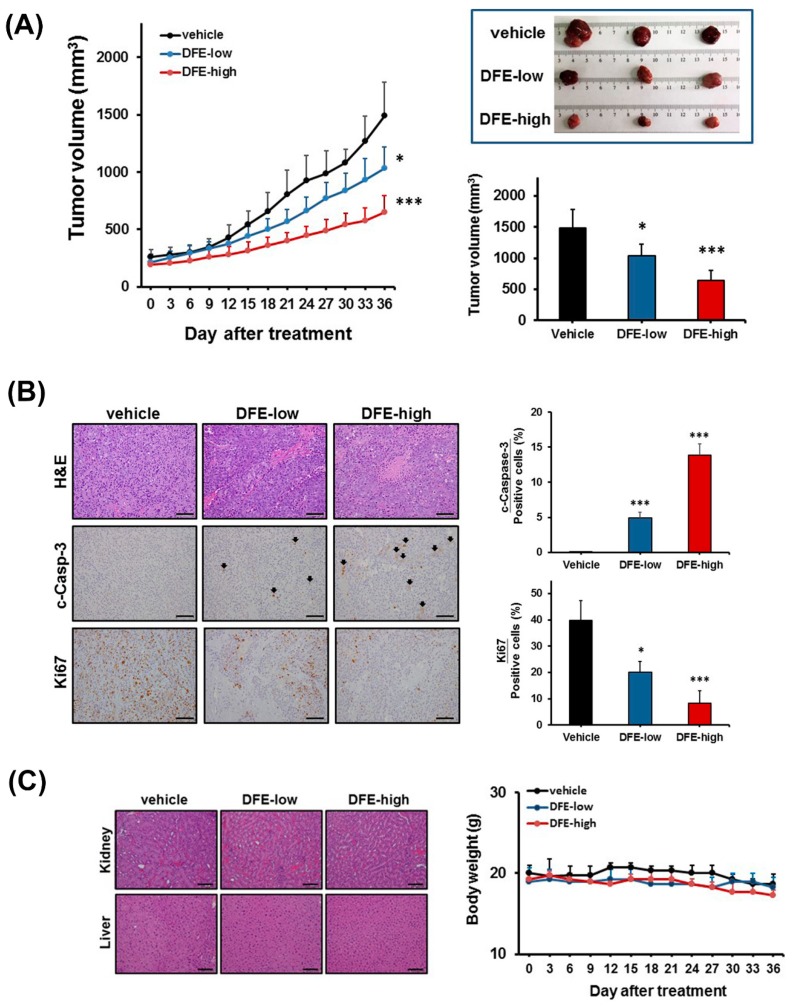
DFE displays anti-PCa activity in vivo. (**A**) DFE (250 (low) and 500 (high) mg/kg body weight) significantly inhibited the growth of subcutaneous C4-2 tumors compared to the control-vehicle group for 36-day observation. The tumor volumes (mm^3^) showed as the mean ± SD (*n* = 5/group). * *p* < 0.05, *** *p* < 0.001. Photographs of representative tumors in each group (Day 36) were shown (top-right panel). (**B**) Immunohistochemical (IHC) staining showed that significant increase of cleaved (c)-caspase-3 (c-Casp-3) and decrease of Ki67 were found in the DFE-treated C4-2 tumors compared to the vehicle groups. Scale bar = 100 μm. Quantifications of c-Caspase-3 and Ki67 were analyzed by the positive stained cell counting in an average of five various fields. *p < 0.05, ***p < 0.001. (**C**) Hematoxylin & Eosin (H&E) staining of both the kidney and liver specimens harvested from the vehicle and the DFE C4-2 tumor-bearing mice (left panel). Scale bar = 100 μm. Besides, no apparent differences were found in the body weights among these three groups during the 36-day treatment (right panel).

To evaluate the anti-PCa potential of DFE in vivo, we first established a subcutaneous xenograft mouse model of C4-2 tumors, castration-resistant PCa cells. Subsequently, the various doses of DFE (250 (low) and 500 (high) mg/kg body weight) or vehicle were given by oral administration daily in the C4-2 tumor-bearing mice, respectively. As shown in Figure 6A (left and right panels), the mice treated with DFE displayed a significant efficacy on volume reduction of C4-2 tumors in a concentration-dependent manner compared to the vehicle control group during 36-day observation. Administration with the DFE-high group significantly attenuated C4-2 tumor volumes from the 12^th^ day onwards (*p* < 0.05) in comparison with the control group. Besides, the DFE-low group showed significant inhibition of the tumor volumes from the 21st day onwards (*p* < 0.05) in comparison with the control group. To determine the underlying molecular basis by which DFE inhibited the C4-2 tumor burdens in mice, immunohistochemical (IHC) staining of c-caspase-3 (a marker of apoptosis) and Ki67 (a marker of cell proliferation) in C4-2 tumor specimens collected from the DFE-treated and the vehicle control mice was performed. Significantly increased c-caspase-3 expression and decreased Ki67 expression in the DFE-treated C4-2 tumors compared to the vehicle group were detected (Figure 6B, left and right panels). The IHC results indicate that DFE reduced cell proliferation and induced caspase-dependent apoptosis in C4-2 tumors in vivo. Moreover, the histopathological features of the kidney and liver specimens were examined to evaluate the general cytotoxicity of DFE treatment in C4-2 xenograft mice. Microscopically, no substantial changes were observed in both kidney and liver tissues harvested from the DFE-low and DFE-high-treated mice in comparison with the vehicle-treated mice (Figure 6C, left panel). Besides, no apparent differences were found in the body weights among these three groups (Figure 6C, right panel). The results of histopathological features and body weights suggest that DFE treatment showed no apparent cytotoxicity in the mice bearing with C4-2 tumors. The in vivo data collectively demonstrated that DFE suppressed growth and induced apoptosis in subcutaneous C4-2 tumors without showing significant cytotoxicity in mice. It indicates that DFE would be able to be employed as a safe and attractive anti-PCa drug. 

## 3. Discussion

*Davallia formosana* is one of the native Taiwanese ferns and has been employed as Chinese medicine to effectively treat skeletal disorders for a long time, but not yet in cancer-associated diseases. In the present paper, we reveal the first time that DFE exerts a promising anticancer activity on clinically related PCa cell lines, LNCaP androgen-dependent and C4-2 castration-resistant cells, through coordinated blockade of SREBP-1/FASN/lipogenesis and the AR axis.

The roles of lipogenesis have gained particular interest in PCa research [10,16,29]. Upregulation of de novo lipogenesis has been delineated to be the increased needs for the fundamental components of lipid-bilayer cell membranes and activation of the lipid raft membrane-controlled signal transduction during rapid and unrestrained cell division, proliferation and aggressiveness in PCa [10,29,30]. Furthermore, PCa cells, in contrast to normal prostatic cells, activate intracellular lipogenesis to be independent of the circulating fatty acids and lipids from diet [31]. Besides, expression of two key protein factors controlling de novo lipogenesis, SREBP-1 and FASN, was highly elevated in clinical prostate cancer and CRPC specimens compared to normal/non-tumor prostate tissues [11,16,19,20]. Thus, targeting the SREBP-1/FASN/lipogenesis network, a PCa-specific metabolic vulnerability, could be exploited therapeutically. In this research, we provided evidence that DFE attenuated the genotypes (Figure 3) and phenotypes (Figure 4) of lipogenesis in PCa cells. DFE reduced the expression of SREBP-1 and FASN mRNA as well as protein in both LNCaP and C4-2 cells. Through reduction of SREBP-1 and FASN, DFE decreased the amounts of intracellular fatty acids and the accumulation of lipid droplets in PCa cells. Down-regulation of SREBP-1 and/or FASN using sequence-specific miRNAs (miRNA-185 and 342 [26]) or selective small compounds (IPI-9119 [32], orlistat [33], and fatostatin [34]) has been reported to inhibit cell growth, survival and progression in cancers. Consistent with our results, through co-inhibition of SREBP-1 and FASN expression as well as reduction of lipogenesis, DFE suppressed growth and decreased migration and invasion in PCa cells (Figure 1 and Figure 2). Besides DFE also decreased expression of cyclin A and cyclin D1 in PCa cells (Figure S1), which are two regulators for cell growth by controlling the cell cycle. Therefore, DFE would be able to be employed as an attractive and novel agent by targeting the SREBP-1/FASN/lipogenesis network to impair unrestrained growth and progression in PCa.

Currently, there is no effective therapy to cure CRPC aggressiveness in patients, which is one of the most challenging clinical issues in the PCa study. Up-regulation of AR expression and activation of the androgen/AR-mediated signaling axis have been well delineated to promote PCa development, survival and fatal CRPC progression [4,5]. Inhibition of androgen biosynthesis, silence of AR expression or interruption of the androgen/AR-associated signaling pathways would be able to offer promising therapeutic strategies to treat PCa and its advanced disease. Next-generation AR-targeted agents have been developed and represent breakthroughs in the treatment of CRPC, including abiraterone (an inhibitor of androgen biosynthesis), enzalutamide and apalutamide (selective antagonists of AR). Nevertheless, the majority of patients who take these new drugs experience transiently clinical and survival benefits. The disease eventually relapses and develops resistance to the next-generation agents [7]. Therefore, seeking alternative therapeutic approaches or finding better drugs to effectively treat this lethal progression are urgent needs. Notably, new research provides significant evidence that blockade of de novo lipogenesis using specific inhibitors of SREBP-1 or FASN could potentially overcome fatal CRPC or drug-resistant PCa aggressiveness [16,32,35]. This is the first research article revealing that DFE concertedly impaired SREBP-1/FASN/lipogenesis and inhibited AR/PSA expression in androgen-dependent as well as castration-resistant PCa cells in vitro and in vivo. By co-targeting SREBP-1/FASN/lipogenesis and the AR axis in PCa, it will be further warranted to investigate the therapeutic efficacy of DFE as an innovative remedy for eradication of CRPC progression and drug-resistant aggressiveness in PCa patients.

Results from this paper indicate that caspase-3 and PARP are involved in DFE-induced apoptosis in PCa cells. A concentration-related increase of the apoptotic cell populations via induction of caspase-3 catalytic activity in PCa cells treated with DFE was observed (Figure 5A,B). Additionally, DFE induced the cleaved forms of caspase-3 and PARP expression in both LNCaP and C4-2 cells determined by Western blotting (Figure 5C). Apoptosis is characterized as an essential biological form of programmed cell death that appears in normal cell turnover and tissue homoeostasis, embryogenesis, cell aging, and cancer therapy [36,37]. Anti-cancer drugs or therapeutic approaches that can induce the cellular apoptotic pathways, including the extrinsic and the intrinsic pathways, have the potential to eradicate cancer cells during programmed cell death [22,23,26]. The extrinsic/death receptor-mediated and the intrinsic/mitochondrial-mediated apoptotic pathways converge at the activation of caspase-3 in cells [38]. Upon the induction of caspase-3 catalytic function, substrates such as PARP are cleaved, eventually resulting in programmed cell death. Therefore, induction of caspase-3 and PARP by DFE leads to the concomitant execution phase of apoptosis in PCa cells in vitro (Figure 5) as well as in mouse xenograft C4-2 tumors in vivo (Figure 6B).

Several bioactive components extracted from DFE have been identified, including davallic acid [39], flavan-3-ol, and proanthocyanidin allosides [40] as well as (−)-epicatechin 3-O-β-D-allopyranoside [41,42]. Among these identified components, (−)-epicatechin 3-O-β-D-allopyranoside has been demonstrated to show anti-inflammatory activity [1], and prevent diabetes and dyslipidemia via alternation of the AMPK and Akt pathways exhibiting low cytotoxicity in the hosts [41,42]. Additionally, we examined this compound in PCa cells. However, (−)-epicatechin 3-O-β-D-allopyranoside (up to 350 μM) didn’t significantly inhibit cell growth in both LNCaP (*p* = 0.173, at 48 h) and C4-2 (*p* = 0.295, at 48 h) cells. Thus, we are attempting to isolate and identify the bioactive ingredients from DFE, which could display cell growth inhibition and apoptotic activity via interruption of SREBP-1/FASN/lipogenesis and the AR axis in PCa cells. Once the bioactive factors are obtained, we will further determine the molecular mechanism by which the bioactive factors extracted from DFE regulate the gene expression of AR, SREBP-1 and FASN in PCa cells. Moreover, these isolated compounds will be also evaluated the efficacy on the metastatic PCa cells, including bone metastatic PC-3 cells [43] and brain metastatic DU145 cells [44], in the future.

Overall, we uncovered that: (1) DFE, a native Taiwanese fern and a Chinese medicine, impaired growth and progression (migration and invasion) in both LNCaP androgen-dependent cells as well as C4-2 castration-resistant cells; (2) DFE significantly diminished the mRNA and protein expression of SREBP-1, FASN, AR, and PSA in PCa cells. The results of gene expression suggest that DFE could lead to coordinated blockade of SREBP-1/FASN/lipogenesis and the AR/PSA axis in PCa cells; (3) By inhibition of SREBP-1 and FASN expression, DFE reduced the intracellular levels of fatty acids and lipid droplet accumulation in PCa; and (4) DFE led to caspase-dependent apoptosis in PCa cells in vitro as well as in mouse xenograft CRPC C4-2 tumors in vivo. In summary, our study provides pre-clinical evidence and a promising therapeutic opportunity that DFE could be potentially applied as an effective pharmacologic management with low cytotoxicity against aggressive PCa.

## 4. Materials and Methods

### 4.1. Cell Lines and Cell Culture Condition

PCa cell lines [25], LNCaP (androgen-dependent), and C4-2 (androgen-refractory/castration-resistant), were provided by Dr. Leland W.K. Chung (Cedars-Sinai Medical Center, Los Angeles, CA, USA). These cells were cultured in Roswell Park Memorial Institute (RPMI) 1640 medium (Thermo Fisher Scientific/GIBCO, Waltham, MA, USA) supplemented with 10% fetal bovine serum (FBS; GE Healthcare/Hyclone, Pittsburgh, PA, USA), 100 U/mL penicillin, and 100 μg/mL streptomycin.

### 4.2. Preparation of Davallia formosana Ethanol Extract (DFE) 

*Davallia formosana* (DF) were purchased from a local market in Taichung, Taiwan. The botanical identification was conducted by the Institute of Chinese Pharmaceutical Sciences, China Medical University, where the voucher specimens were deposited. Briefly, the rhizomes of DF (1000 g) were extracted with 50% ethanol (10 L). Subsequently, the mixtures of DF were filtered through filter paper, and the residues were extracted with 50% ethanol for one more time. The filtrates were collected together and subjected to concentration under reduced pressure to produce DF extract (DFE). The yield of DFE was approximately 25%. DFE was stored at −80 °C until use.

### 4.3. Analyses of PCa Cell Growth, Migration, and Invasion

For cell growth analysis [34], PCa cells were seeded in 96-well plates (1 × 10^4^ cells/well) for overnight and subsequently treated with various concentrations (10, 20, 40, 80, and 160 µg/mL) of DFE or vehicle (0.06% DMSO). Cell growth was determined by MTT assay (Promega) according to the manufacturer’s instructions. A wound healing analysis was performed to measure cell motility. Briefly, PCa cells were grown on a confluent monolayer in a 6-well cell culture dish. Cell monolayers were “wounded” using a P200 micropipette tip. The wounded monolayers were then washed twice with phosphate-buffered saline (PBS) to remove cell debris and incubated in cultured medium with DFE (20, 40 and 80 µg/mL) or vehicle (0.06% DMSO). The migrating cells were recorded by an inverted microscope equipped with a camera for 48 and 96 h after the treatment of reagents. Additionally, the migratory and invasive capabilities were determined by the transwell chambers pre-coated with nothing (for migration assay) or growth factor-depleted Matrigel matrix (BD Bioscience, San Jose, CA, USA; for invasion assay) [26,28]. PCa cells (1 × 10^5^ cells/well) were seeded into the inside of the pre-coated upper chambers and then treated with DFE (20, 40 and 80 µg/mL) or vehicle (0.06% DMSO). The migrated or invaded cells were fixed with 100% methanol and stained using 2% crystal violet in methanol after 48 h treatment. Subsequently, a light microscope was used to count the migrated or invaded PCa cells in randomly selected fields.

### 4.4. Quantitative Reverse Transcription-Polymerase Chain Reaction (RT-qPCR)

Total RNA samples were prepared from vehicle- or DFE (20 µg/mL)-treated LNCaP and C4-2 cells by REzol C & T (Protech Technology, Taipei, Taiwan) and reversely converted to cDNA using iScript cDNA Synthesis Kit (Bio-Rad, Hercules, CA, USA). The qPCR analysis was conducted using iQ SYBR Green Supermix (Bio-Rad) and a CFX96 Touch Real-Time PCR Detection System (Bio-Rad). The sequences of the oligonucleotide primers used for qPCR analysis were listed in Table S1, including AR, PSA, FASN, SREBP-1, SREBP-2, HMGCR, and β-actin. Data were normalized to internal control β-actin and represented as the average ratio of triplicates.

### 4.5. Western Blotting 

Total protein samples were extracted from vehicle- or DFE (20 µg/mL)-treated PCa cells by PRO-PREP Protein Extraction Solution (iNtRON technology, Seoul, South Korea) adding with protease inhibitors. A Pierce^TM^ BCA Protein Assay Kit (Thermo Fisher Scientific) was utilized to determine the concentrations of proteins. The equal amounts of protein samples were loaded into SDS-PAGE gels for electrophoresis and then proteins were transferred onto polyvinylidene difluoride (PVDF) membranes. Subsequently, the blotted membranes were blocked using 5% non-fat milk in PBS with Tween-20. After blocking, the membranes were incubated with primary antibodies for overnight, followed by incubation with a horseradish peroxidase-conjugated secondary antibody. Primary antibodies were used in this study as follow: anti-AR, anti-SREBP-1, anti-FASN (Santa Cruz Biotechnology, Dallas, TX, USA), anti-SREBP-2 (abcam, Cambridge, MA, USA), anti-caspase-3 (Novus Biologicals, Littleton, CO, USA), anti-PARP (GeneTex, Irvine, CA, USA), and anti-β-actin (Millipore, Burlington, MA, USA). The protein signals were visualized using an Enhanced Chemiluminescence Kit (Amersham Biosciences, Arlington Heights, IL, USA) and an imaging system (ImageQuant LAS 4000; GE Healthcare, Pittsburgh, PA, USA). The ImageJ software was utilized to quantify specific protein bands by normalized a loading control, β-actin.

### 4.6. Assays of Lipogenesis

PCa cells treated with vehicle or various concentrations of DFE were assayed the contents of long chain fatty acids by a Fatty Acid Quantification Kit (MBL International Corporation, Woburn, MA, USA) according to the manufacturer’s instructions. In addition, an Oil Red O staining method [16] was used to determine lipid droplet formation in PCa cells. Oil Red O staining images of PCa cells treated with vehicle or DFE were examined and recorded by a phase contrast microscope. For quantification of lipid droplet formation, Oil Red O retained in PCa cells was extracted by isopropanol and subsequently determined by optical absorbance at 500 nm. Data were normalized by total cell numbers or total protein concentrations.

### 4.7. Flow Cytometric Analysis of Apoptosis 

PCa cells were treated with vehicle or various concentrations (20, 40, 80, and 160 μg/mL) of DFE for 24 h. Subsequently, these cells were stained by a FITC-Annexin V Apoptosis Detection Kit with propidium iodide (PI) (Biolegend, San Diego, CA, USA) according to the manufacturer’s instruction. The apoptotic cells (%) were analyzed and calculated by flow cytometry (BD FACSCanto, BD Bioscience) and a FACS Express v2.0 software.

### 4.8. Mouse Experiments

Mouse experiments were performed in accordance with the protocol approved by the Institution Animal Care and Use Committee (IACUC) of China Medical University (protocol No. 2018-029). All mice were maintained under standard pathogen-free conditions and cared for, according to the criteria outlined in the National Academy of Sciences Guide for the Care and Use of Laboratory Animals. Four-week-old nude male mice (BALB/cAnN.Cg-Foxn1nu/CrlNarl) were purchased from the National Laboratory Animal Center (Taipei, Taiwan) and subcutaneously implanted with C4-2 cells (1 × 10^6^ cells). Mice bearing C4-2 tumors with approximately 100–150 mm^3^ in volume were randomly divided into three groups (*n* = 5 for each group): vehicle control group, DFE-low group (250 mg/kg body weight), and DFE-high group (500 mg/kg body weight; the used doses referred to the previous study and made modifications [45]) with daily feeding by oral gavage for 36 days. Both of the tumor volumes and the body weights were monitored every 3 days. At the end of the mouse experiments (after 36-day treatment), all mice were euthanized using CO_2_ and subcutaneous C4-2 tumors were excised. IHC staining was conducted on the tumor samples for the analyses of cleaved caspase-3 (apoptosis) and Ki67 (cell proliferation) [34,46]. 

### 4.9. Statistical Analysis 

All data were analyzed at least three individual experiments by using two-tailed unpaired Student’s *t* test for comparison of independent means. *p* values of less than 0.05 were considered statistically significant. * *p* < 0.05, ** *p* < 0.01, *** *p* < 0.001.

## 5. Conclusions

Our findings offer novel insight into the underlying molecular basis of DFE in androgen-dependent and castration-resistant PCa cells in vitro and in vivo. Co-targeting SREBP-1/FASN/lipogenesis and the AR/PSA axis by DFE will be able to be developed as a new and promising remedy to cure aggressive PCa.

## Supplementary Materials

The following are available online at https://www.mdpi.com/2072-6694/12/4/914/s1, Figure S1: DFE reduced cyclin A and cyclin D1 expression in PCa cells, Table S1: The oligonucleotide primer sets and sequences for qPCR analysis, The uncropped blots and molecular weight markers are shown in Supplementary Materials.
