# Supplementary material for: Emerging Therapeutic Activity of Davallia formosana on Prostate Cancer Cells through Coordinated Blockade of Lipogenesis and Androgen Receptor Expression
Source: Cancers (Basel). 2020 Apr 8;12(4):914. doi: 10.3390/cancers12040914 (PMC7226131; doi:10.3390/cancers12040914)
Supplement: Supplementary file 1 [file cancers-12-00914-s001.zip › Supplementary Data/Figure S1.docx]

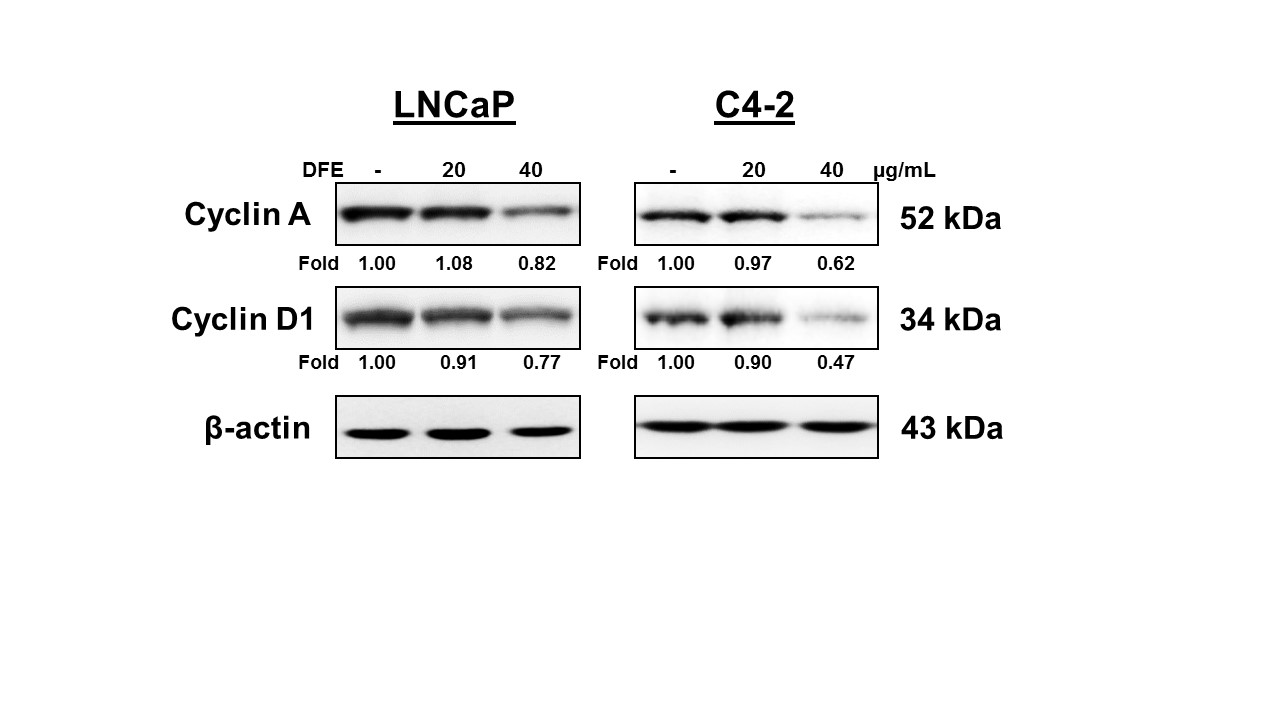


**Figure S1.** DFE reduced cyclin A and cyclin D1 expression in PCa cells. β-actin was used as a loading control. The relative fold was defined as 1.00 in the vehicle-treated (-) LNCaP and C4-2 cells, respectively. Data were normalized by β-actin.
