# Supplementary material for: Emerging Therapeutic Activity of Davallia formosana on Prostate Cancer Cells through Coordinated Blockade of Lipogenesis and Androgen Receptor Expression
Source: Cancers (Basel). 2020 Apr 8;12(4):914. doi: 10.3390/cancers12040914 (PMC7226131; doi:10.3390/cancers12040914)
Supplement: Supplementary file 1 [file cancers-12-00914-s001.zip › Supplementary Data/Supplementary Table S1.docx]

| **Gene** | **Forward (5’-3’)** | | **Reverse (5’-3’)** |
| --- | --- | --- | --- |
| AR [1,2] | GCCTTGCTCTCTAGCCTCAA | GTCGTCCACGTGTAAGTTGC | |
| PSA [3,4] | GTGTGTGGACCTCCATGTTATT | CCACTCACCTTTCCCCTCAAG | |
| FASN [5,6] | ACAGCGGGGAATGGGTACT | GACTGGTACAACGAGCGGAT | |
| SREBP-1 [7,8] | ACAGTGACTTCCCTGGCCTAT | GCATGGACGGGTACATCTTCAA | |
| SREBP-2 [2] | CCCCTGACTTCCCTGCTGCA | GCGCGAGTGTGGCCGGATC | |
| HMGCR [2] | GTCATTCCAGCCAAGGTTGT | GGGACCACTTGCTTCCATTA | |
| β-actin [9,10] | AACTGGAACGGTGAAGGTGAC | TGTGGACTTGGGAGAGGACTG | |

**Supplemental Table S1.** The oligonucleotide primer sets and sequences for qPCR analysis.

**References**

1. Mukhopadhyay, N.K.; Kim, J.; You, S.; Morello, M.; Hager, M.H.; Huang, W.C.; Ramachandran, A.; Yang, J.; Cinar, B.; Rubin, M.A., et al. Scaffold attachment factor B1 regulates the androgen receptor in concert with the growth inhibitory kinase MST1 and the methyltransferase EZH2. *Oncogene* **2014**, *33*, 3235-3245.

2. Li, X.; Chen, Y.T.; Josson, S.; Mukhopadhyay, N.K.; Kim, J.; Freeman, M.R.; Huang, W.C. MicroRNA-185 and 342 inhibit tumorigenicity and induce apoptosis through blockade of the SREBP metabolic pathway in prostate cancer cells. *PLoS One* **2013**, *8*, e70987.

3. Lim, S.; Lee, W.; Lee, D.S.; Nam, I.J.; Yun, N.; Jeong, Y.; Rho, T.; Kim, S. Botanical Formulation HX109 Ameliorates TP-Induced Benign Prostate Hyperplasia in Rat Model and Inhibits Androgen Receptor Signaling by Upregulating Ca(2+)/CaMKKbeta and ATF3 in LNCaP Cells. *Nutrients* **2018**, *10*.

4. Li, H.; Pham, T.; McWhinney, B.C.; Ungerer, J.P.; Pretorius, C.J.; Richard, D.J.; Mortimer, R.H.; d'Emden, M.C.; Richard, K. Sex Hormone Binding Globulin Modifies Testosterone Action and Metabolism in Prostate Cancer Cells. *Int J Endocrinol* **2016**, *2016*, 6437585.

5. Muse, E.D.; Yu, S.; Edillor, C.R.; Tao, J.; Spann, N.J.; Troutman, T.D.; Seidman, J.S.; Henke, A.; Roland, J.T.; Ozeki, K.A., et al. Cell-specific discrimination of desmosterol and desmosterol mimetics confers selective regulation of LXR and SREBP in macrophages. *Proc Natl Acad Sci U S A* **2018**, *115*, E4680-E4689.

6. Guijas, C.; Perez-Chacon, G.; Astudillo, A.M.; Rubio, J.M.; Gil-de-Gomez, L.; Balboa, M.A.; Balsinde, J. Simultaneous activation of p38 and JNK by arachidonic acid stimulates the cytosolic phospholipase A2-dependent synthesis of lipid droplets in human monocytes. *J Lipid Res* **2012**, *53*, 2343-2354.

7. Yang, X.; Wu, F.; Chen, J.; Wang, C.; Zhu, Y.; Li, F.; Hao, Q.; Duan, C.; Wang, L.; Ma, X., et al. GP73 regulates Hepatic Steatosis by enhancing SCAP-SREBPs interaction. *Sci Rep* **2017**, *7*, 14932.

8. Cheng, Y.; Huang, L.; Ping, J.; Chen, T.; Chen, J. MicroRNA-199a-3p attenuates hepatic lipogenesis by targeting Sp1. *Am J Transl Res* **2017**, *9*, 1905-1913.

9. Mayas, M.D.; Ortega, F.J.; Macias-Gonzalez, M.; Bernal, R.; Gomez-Huelgas, R.; Fernandez-Real, J.M.; Tinahones, F.J. Inverse relation between FASN expression in human adipose tissue and the insulin resistance level. *Nutr Metab (Lond)* **2010**, *7*, 3.

10. Macias-Gonzalez, M.; Cardona, F.; Queipo-Ortuno, M.; Bernal, R.; Martin, M.; Tinahones, F.J. PPARgamma mRNA expression is reduced in peripheral blood mononuclear cells after fat overload in patients with metabolic syndrome. *J Nutr* **2008**, *138*, 903-907.
