# Supplementary figures and images for: Emerging Therapeutic Activity of Davallia formosana on Prostate Cancer Cells through Coordinated Blockade of Lipogenesis and Androgen Receptor Expression
Source: Cancers (Basel). 2020 Apr 8;12(4):914. doi: 10.3390/cancers12040914 (PMC7226131; doi:10.3390/cancers12040914)

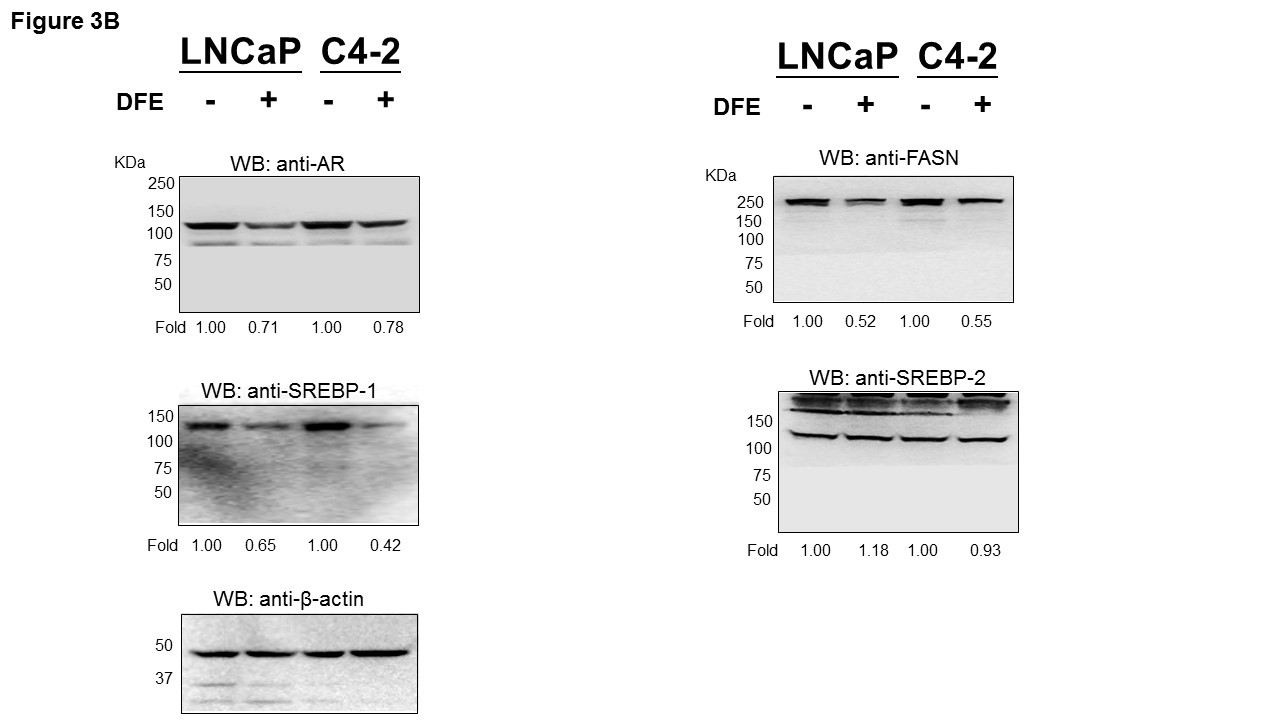


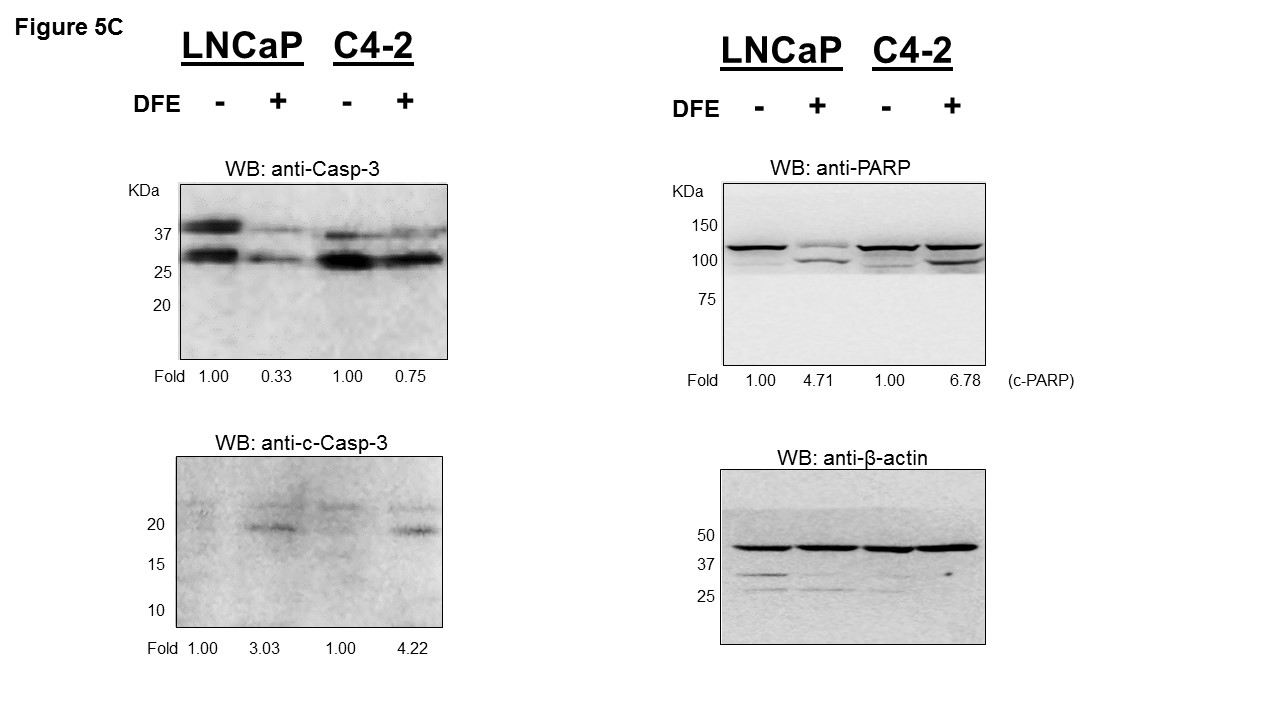


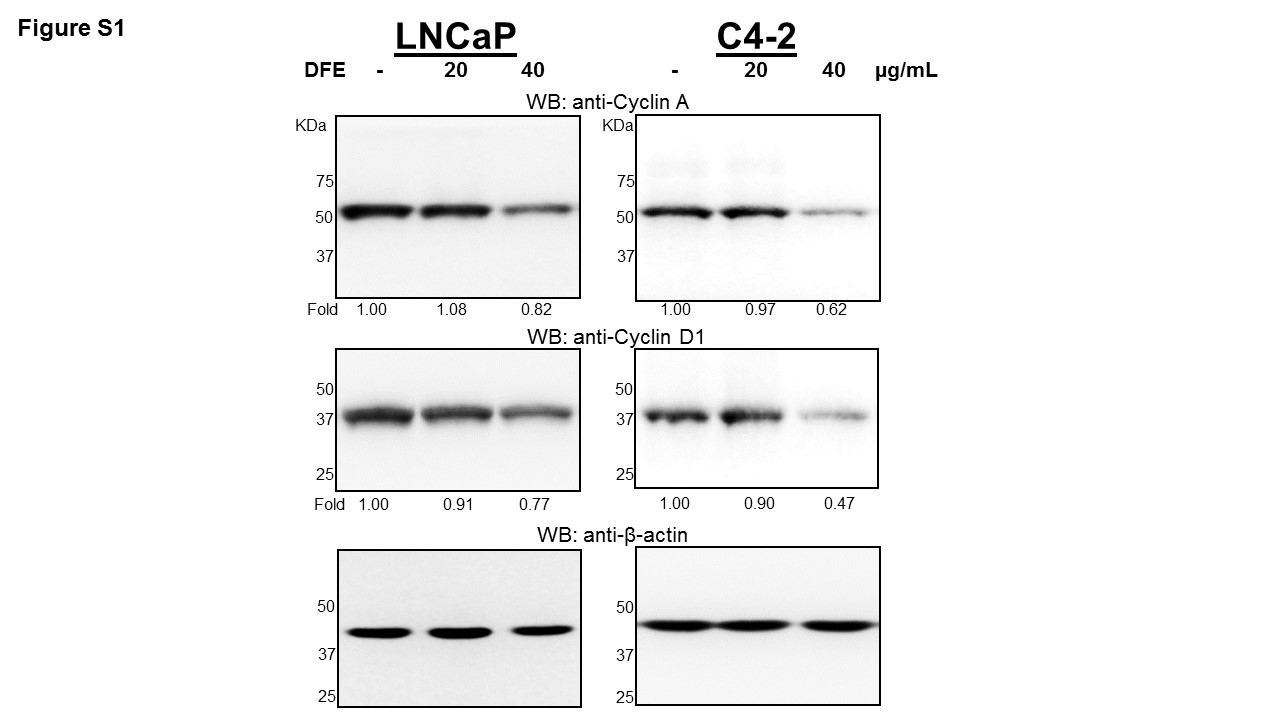

Supplement: Supplementary file 1 [file cancers-12-00914-s001.zip › Supplementary Data/Western blot analysis.docx]
